# Autophagy dysregulation in Danon disease

**DOI:** 10.1038/cddis.2016.475

**Published:** 2017-01-19

**Authors:** Anna Chiara Nascimbeni, Marina Fanin, Corrado Angelini, Marco Sandri

**Affiliations:** 1 Department of Neurosciences, Biomedical Campus Pietro d'Abano, University of Padova, Padova, Italy; 2 Institut Necker Enfant-Malades (INEM), INSERM U1151-CNRS UMR 8253, Université Paris Descartes-Sorbonne Paris Cité, Paris, France; 3 Fondazione IRCCS San Camillo Hospital, Venice, Italy; 4 Venetian Institute of Molecular Medicine, Padova, Italy; 5 Department of Biomedical Science, Venetian Institute of Molecular Medicine (VIMM), University of Padova, Padova, Italy

## Abstract

The autophagy–lysosome system is critical for muscle homeostasis and defects in lysosomal function result in a number of inherited muscle diseases, generally referred to as autophagic vacuolar myopathies (AVMs). Among them, Danon Disease (DD) and glycogen storage disease type II (GSDII) are due to primary lysosomal protein defects. DD is characterized by mutations in the lysosome-associated membrane protein 2 (*LAMP2*) gene. The DD mouse model suggests that inefficient lysosome biogenesis/maturation and impairment of autophagosome-lysosome fusion contribute to the pathogenesis of muscle wasting. To define the role of autophagy in human disease, we analyzed the muscle biopsies of DD patients and monitored autophagy and several autophagy regulators like transcription factor EB (TFEB), a master player in lysosomal biogenesis, and vacuolar protein sorting 15 (VPS15), a critical factor for autophagosome and endosome biogenesis and trafficking. Furthermore, to clarify whether the mechanisms involved are shared by other AVMs, we extended our mechanistic study to a group of adult GSDII patients. Our data show that, similar to GSDII, DD patients display an autophagy block that correlates with the severity of the disease. Both DD and GSDII show accumulation and altered localization of VPS15 in autophagy-incompetent fibers. However, TFEB displays a different pattern between these two lysosomal storage diseases. Although in DD TFEB and downstream targets are activated, in GSDII patients TFEB is inhibited. These findings suggest that these regulatory factors may have an active role in the pathogenesis of these diseases. Therapeutic approaches targeted to normalize these factors and restore the autophagic flux in these patients should therefore be considered.

The autophagic pathway has a crucial role in skeletal muscle homeostasis, providing a finely tuned system for protein degradation and organelle removal.^1^ Macro-autophagy, hereafter referred to as autophagy, occurs through the formation of specific organelles called autophagosomes, which engulf organelles, cellular cytoplasm and proteins aggregates and fuse with lysosomes for the cargo degradation. Lysosomes are the final effectors of this process and their importance in muscle is highlighted by a group of muscle diseases, characterized by a massive buildup of autophagic vacuoles and by abnormal lysosomes. Due to the tremendous accumulation of autophagosomes, these disorders have been referred to as autophagic vacuolar myopathies (AVMs) and include X-linked myopathy with excessive autophagy (XMEA), Danon disease (DD) and Pompe/glycogen storage disease type II (GSDII).^2^ The genes that cause AVMs encode for proteins involved in lysosomal acidification, in lysosomal degradation of glycogen and in the maturation and fusion of autophagosomes with lysosomes. Knockout mice for autophagy genes have shown that autophagy impairment is detrimental for myofiber health and is potentially involved in the pathogenesis of these conditions.^3^ However, little is known about autophagy status and regulation in patients.

DD is an X-linked dominant disorder caused by the deficiency of lysosome-associated membrane protein 2 (LAMP2).^4^ DD patients typically show hypertrophic cardiomyopathy, muscle weakness and mental retardation.^5^ LAMP2 is mainly localized in the limiting membranes of lysosomes and late endosomes and, in small amounts, in early endosomal and plasma membrane. LAMP2 is required for the maturation of early autophagosomes, which involves an intermediate fusion step with endosomes. The mouse model of the disease shows a similar vacuolar degeneration of cardiomyocytes and myofibers resulting in a cardiomyopathy and myopathy.^4^ In mice, absence of LAMP2 blocks the normal maturation of autophagosomes. Indeed, the rodent LAMP2-deficient hepatocytes exhibit accumulation of early autophagic vacuoles, mistargeting of lysosomal enzymes including LAMP1, improper cathepsin D processing, abnormal retention of mannose-6-phosphate receptors in autophagic vacuoles, reduction of degradation of long-lived proteins and resistance to autophagy-dependent protein breakdown during starvation.^6^

*LAMP2* gene has three isoforms: *LAMP2a*, *LAMP2b* and *LAMP2c*. LAMP2a functions as a receptor for chaperone-mediated autophagy (CMA). CMA is a proteolytic system in which proteins that contain the pentapeptide KFERQ are selectively targeted to the lysosome for degradation, through the coordinated action of chaperones and a dedicated protein translocation complex formed by LAMP2a.^7, 8^ LAMP2b and LAMP2c result from alternative splicing of exon 9. Although the deficiency of LAMP2a-mediated CMA may trigger autophagy as a compensatory mechanism, the half-life of autophagosomes in LAMP2-deficient murine hepatocytes is prolonged, suggesting an impairment of autophagosome–lysosome fusion and degradation.^9^ Muscles of DD patients have large vacuoles surrounded by lysosomes and delimited by membranes containing sarcolemmal proteins (such dystrophin-associated proteins, extracellular matrix molecules and acetylcholinesterase), and positive for the autophagosome marker LC3. These structures are described as ‘autophagic vacuoles with sarcolemmal features' (AVSF).^10^ AVSFs appear to be constituted by an accumulation of autophagosomes, lysosomes and autophagolysosomes. Therefore, the pathogenic mechanism of DD seems to be related to impairment of autophagosome-lysosome fusion and/or to an inefficient lysosome biogenesis and maturation.

GSDII is an autosomal recessive disorder caused by a defect in lysosomal acid α-glucosidase (GAA),^11^ the enzyme that hydrolyzes lysosomal glycogen to glucose. The presence of large glycogen-filled lysosomes and massive autophagic buildup in muscles are hallmarks of GSDII.^12, 13^ Complete GAA deficiency causes the severe form of GSDII with infantile onset (Pompe disease), whereas partial enzyme defects induce late-onset milder forms of the disease, that are mainly characterized by progressive skeletal muscle weakness, while cardiac muscle is spared. We previously demonstrated a correlation between impaired autophagy and myofiber atrophy and weakness in late-onset GSDII.^14^ Autophagy inhibition also compromises the efficacy of enzyme replacement therapy (ERT), the only available therapy for GSDII.^15^ In this study, we extended our observation of autophagy impairment in GSDII to DD and investigated the status of two important autophagy regulators: vacuolar protein sorting 15/phosphoinositide-3-kinase regulatory subunit 4 (VPS15/PIK3R4) and transcription factor EB (TFEB). Furthermore, we compared the level of autophagy activation between DD with GSDII patients.

## Results

### Monitoring autophagy and atrophy in DD muscle

**Figure 1:**
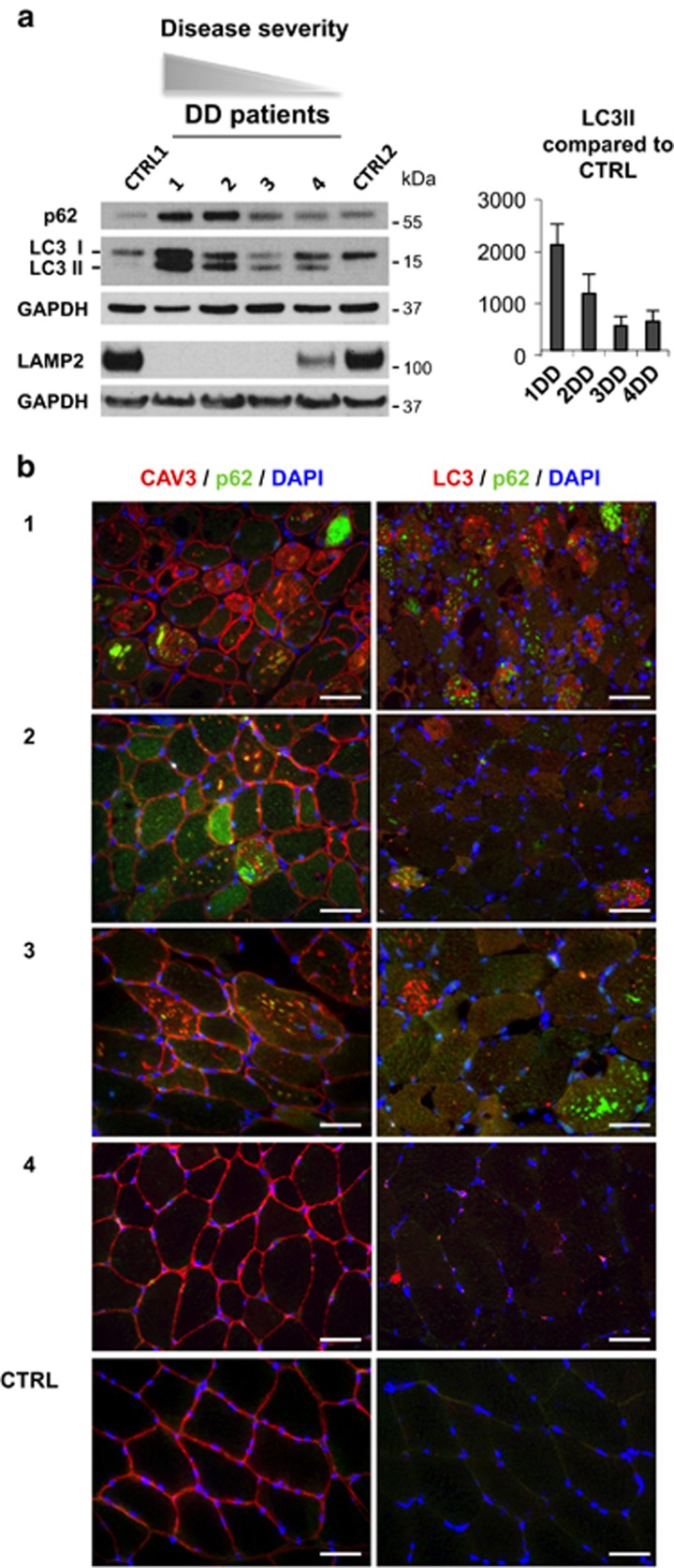
Characterization of autophagy in Danon disease. (**a**) Immunoblot analysis of LC3, p62, LAMP2 and the loading control GAPDH. Ctrl1, Ctrl2, healthy controls; numbers, patient identifiers. LC3II protein quantification normalized to GAPDH and compared with healthy controls. (**b**) Immunofluorescence analysis of muscle biopsies for p62-positive aggregates (green), the sarcolemmal membrane and vacuolar marker CAV3 and LC3 puncta (red), and the nuclear marker DAPI (blue) in DD patients and healthy adult control. Bar=40 *μ*m

To investigate whether autophagy is involved in DD muscle pathology, we studied muscle biopsies of four DD patients (Supplementary Table S1), including three males (with the classical phenotype) and one female (with cardiomyopathy and mild muscle involvement). All male patients (pts. 1, 2 and 3) showed a complete LAMP2 protein deficiency, whereas the female pt. 4 showed a reduced protein amount (Figure 1a). We quantified by immunoblotting the lipidated form of LC3 (LC3II), which is present on autophagosome membranes, and SQSTM1/p62, a substrate of autophagy that reveals how efficient is autophagosome clearance (Figure 1a). Male patients showed a variable increase of LC3II and p62, which correlated with disease progression and muscle pathology. The highest levels were detected in the most severely affected pt. 1 (Supplementary Table S1). The accumulation of LC3II and p62 is consistent with an accumulation of autophagosomes consequent to impaired delivery or fusion with lysosomes. Therefore, we monitored the presence of vacuoles and p62-positive inclusions inside myofibers and quantified the percentage of fibers presenting these features (Figure 1b). Our previous studies in humans and mice have shown that p62-positive protein aggregates are a marker of autophagic flux impairment that can reveal autophagy inhibition in patients' muscle.^3, 14^ We used double immunofluorescence staining for caveolin-3 (CAV3), to mark vacuolar membranes, and for p62, to mark p62-positive aggregates. Importantly, p62-positive inclusions were found both in the cytoplasm and inside the AVSF (Supplementary Figure S1). The female pt. 4 showed levels of p62 and LC3II very similar to controls (Figure 1a) and no vacuoles or p62-positive inclusions (Figure 1b). Conversely, the three male patients showed several p62-positive inclusions. Autophagy impairment was also confirmed by the autophagic buildup revealed by the accumulation of LC3 positive puncta (Figure 1b). Importantly, LC3 did not colocalize with the big p62-positive aggregates, confirming that the staining was not consequent to non-specific sequestration of LC3 into protein aggregates.

**Figure 2:**
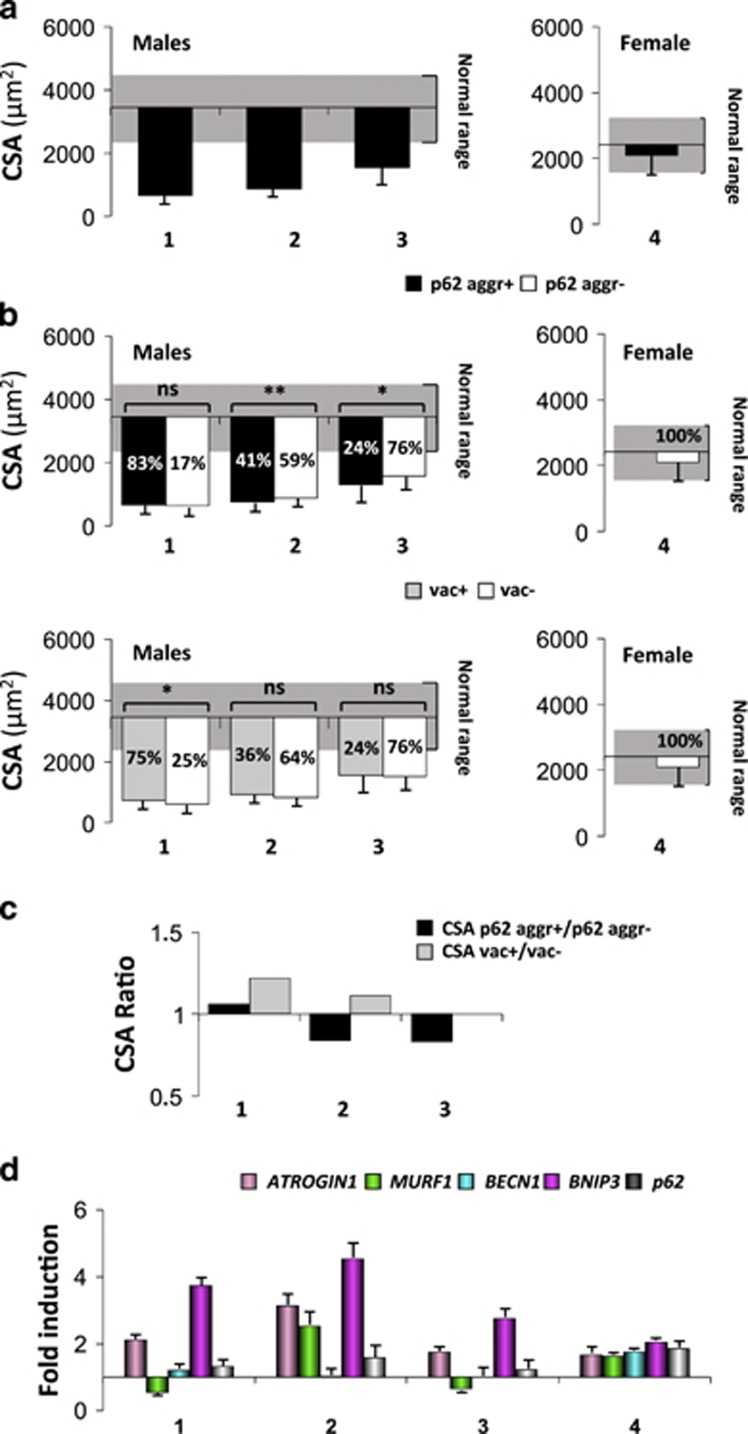
Characterization of atrophy and autophagy in Danon disease. (**a**) Mean CSA of total myofibers. (**b**) CSA of fibers showing p62-positive aggregates (p62+ aggr) compared with fibers without p62-positive aggregates (p62- aggr) and vacuolated (vac+) compared with non-vacuolated (vac-) fibers, relative to healthy age- and sex-matched controls (male controls mean CSA: 3446±950 *μ*m^2^; female controls mean CSA: 2419±786 *μ*m^2^; dashed box: normal CSA range). The percentages of p62-positive, negative, vacuolated and non-vacuolated fibers are reported in the bars. **P<0.0001; *P<0.05; NS, not significant. *n*>200 fibers measured. (**c**) CSA ratio of p62-positive versus negative and vacuolated versus non-vacuolated fibers. When the size of fibers containing p62 aggregates (or vacuoles) is smaller than that of the negatives, it will result in a ratio below 1. (**d**) qRT-PCR analysis of the atrophy-related genes and of p62. The fold induction is compared with age-matched controls and normalized to GAPDH

We measured fibers cross-sectional area (CSA) to assess whether myofiber size is affected when autophagy (p62-positive aggregates) or lysosomes (vacuoles) are compromised. The three male patients showed and important fiber atrophy, while the female patient showed a normal range of fiber size (Figure 2a). The percentage of p62-positive fibers revealed a good correlation with fiber atrophy and the severity of the disease. Indeed, pt. 1 showed p62-positive inclusions in most of the fibers, while pt. 4 had no p62-positive aggregates. We compared the CSA of the fibers that contained p62-positive inclusions with the surrounding p62-negative and of vacuolated versus non-vacuolated fibers. CSA of p62-positive fibers was significantly lower than the surrounding p62-negative in two of the three male patients (Figure 2b), while fiber size did not differ between vacuolated and non-vacuolated fibers, except in pt. 1 (Figure 2b). To better understand the relationship between autophagy, vacuoles and muscle loss, we determined the ratio between the CSA of p62-positive versus p62-negative and of vacuolated versus non-vacuolated fibers. Importantly, a ratio below 1 indicates that the altered fibers are more atrophic than the surrounding normal ones (Figure 2c). The ratio of p62-positive was lower than the ratio of vacuolated fibers in all patients and below 1 in two of three patients indicating that autophagy impairment is detrimental for muscle mass maintenance and contributes to muscle atrophy (Figure 2c). Pt. 1, whose muscle fibers were extremely atrophic, showed a ratio very close to 1. However, the CSA measurement of unaffected fibers may not be representative of the distribution of normal fiber size because very few fibers were clear from p62-positive inclusion.

Since muscle fibers were atrophic we monitored the expression of a subset of atrophy-related genes (atrogenes),^16^ some of which are rate-limiting factors of the ubiquitin-proteasome and autophagy–lysosome systems. The upregulation of these factors is sufficient to increase protein breakdown. Thus, we monitored the expression of atrogenes in muscle biopsies to determine whether they correlate with atrophy. The atrophy-related ubiquitin ligase *ATROGIN1/FBX032* and the autophagy-related gene *BNIP3* (Bcl-2/adenovirus E1B 19 kDa interacting protein 3) were induced (more than twofold) in male patients (Figure 2d), consistenly with the important fiber atrophy present in DD males. Conversely, the female patient showed a minor upregulation (about 1.5-fold) of all these genes.

### VPS15 in DD and GSDII muscle

**Figure 3:**
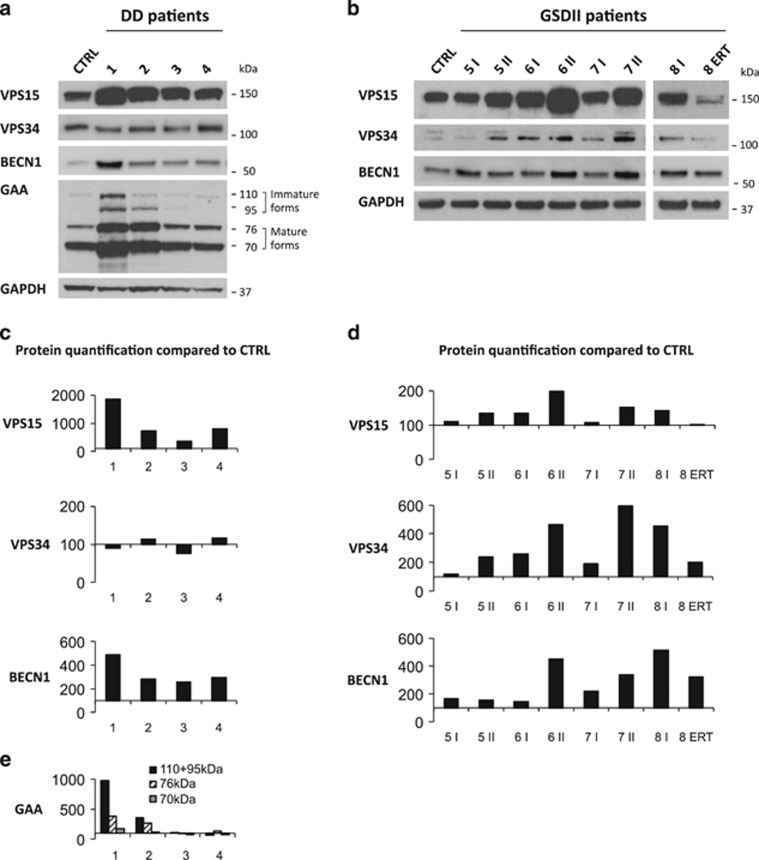
VPS15 expression in DD and GSDII muscle. (**a**) Immunoblot analyses of muscle biopsies for VPS15, VPS34, BECN1, GAA (in DD) and the loading control GAPDH in DD and in GSDII (**b**) patients. Ctrl, healthy control; ERT, biopsy after ERT; numbers, patient identifiers; I, first biopsy; II: second biopsy. (**c**) Densitometric analysis (percentage compared with control) of proteins normalized to GAPDH for DD and GSDII (**d**) patients. (**e**) Densitometric analysis (percentage compared with control) of GAA. The different GAA forms are specified: the inactive (110+95 kDa) and the active ones (76 and 70 kDa)

Initiation of autophagy requires the autophagy-specific class III phosphatidylinositol-3-kinase complex (PI3K), which is constituted by different factors including vacuolar protein sorting 34 (VPS34/ PIK3C3), VPS15, Beclin1/BECN1 and ATG14L.^17^ This complex generates phosphatidylinositol-3-phosphate (PI3P), which allows the recruitment of the autophagy conjugation system to recruit LC3II on the membrane for the autophagosome formation. However, the PI3K complex is also involved in regulation of endosome trafficking. The different actions of VPS34/VPS15 depend on the partners that are recruited in the complex.^18, 19^ VPS15 is a regulatory subunit required for VPS34 stability and activation. In VPS15-deficient muscles the autolysosome clearance is impaired and the muscle phenotype is reminiscent of lysosomal myopathies with glycogen accumulation.^20^ Therefore, to understand whether changes of VPS15 occur in AVMs, we checked its expression in DD and GSDII muscles. Interestingly, VPS15 protein was increased in DD muscles (Figure 3a and c). To better understand whether VPS15 accumulation was peculiar of DD or common to other AVMs, we analyzed GSDII muscle biopsies. We studied three GSDII patients who underwent two muscle biopsies each, the second one after 6 (pts. 6 and 7) and 9 (pt. 5) years from the first biopsy, and one patient who received ERT (pt. 8) and had one pre- and one post-ERT biopsy. All these patients (except the ERT-treated one), showed an important time-dependent deterioration of muscle function (Supplementary Table S1), with a concomitant impairment of autophagy.^14^ Interestingly, the comparison of these two time points showed a progressive accumulation of VPS15 protein in pts. 5, 6 and 7 but not in the ERT-treated one, who conversely displayed an opposite trend (Figure 3b and d). Therefore, these two different AVMs show a correlation between disease progression, autophagy impairment and VPS15 accumulation. The increase of VPS15 is not due to an induction of gene expression (Supplementary Fig. S2) and therefore it is consequent to increased protein half-life. When analyzing the other components of the VPS15 complex we found a similar accumulation for BECN1 and VPS34 in GSDII but not in DD. Indeed, LAMP2-deficient muscles showed an accumulation of BECN1 that matched with VPS15, but no changes of VPS34.

**Figure 4:**
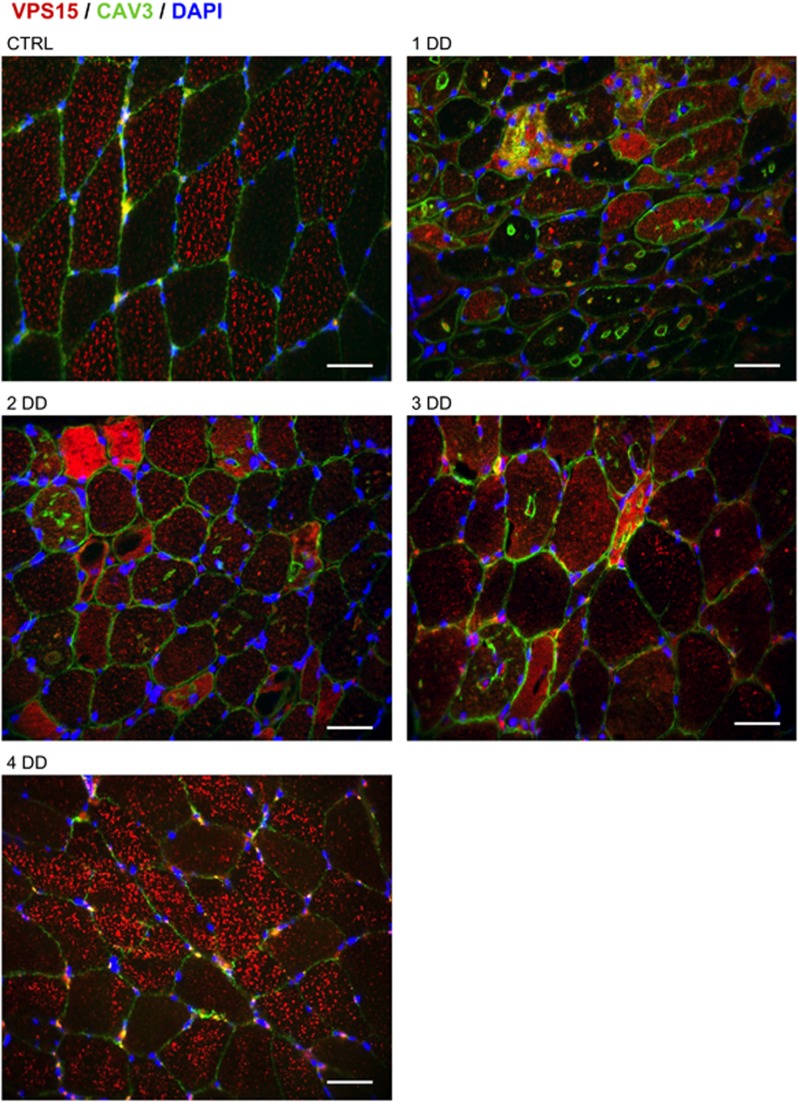
VPS15 localization in DD muscle. Immunofluorescence analysis of muscle biopsies for VPS15 (red), CAV3 (green) and DAPI (blue). Ctrl, healthy control; numbers, patient identifiers. Bar=40 *μ*m

**Figure 5:**
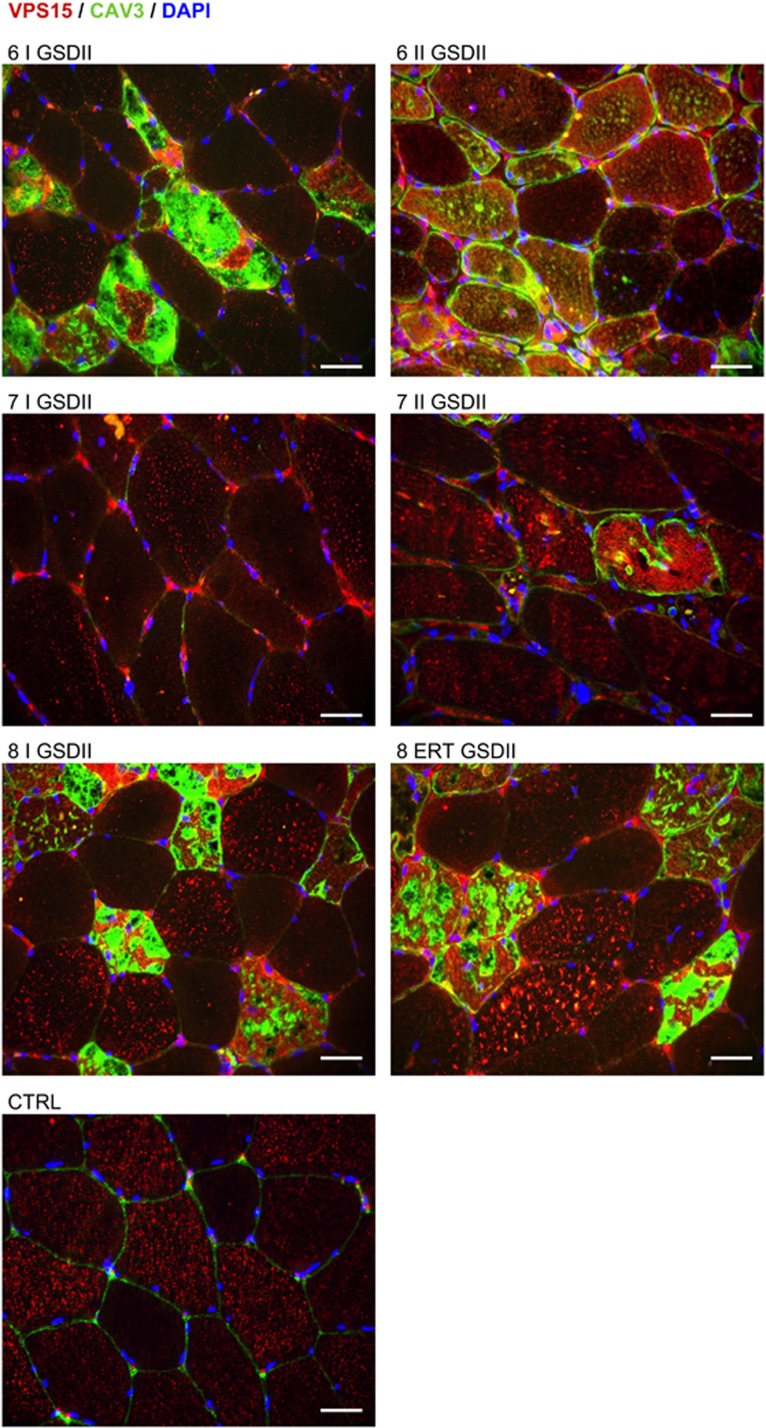
VPS15 localization in GSDII muscle. Immunofluorescence analysis of muscle biopsies for VPS15 (red), CAV3 (green) and DAPI (blue). Ctrl, healthy control; numbers, patient identifiers; I, first biopsy; II, second biopsy; ERT, biopsy after ERT. Bar=40 *μ*m

To understand whether VPS15 increase was associated with a correct cytoplasmic localization, we immunostained muscle biopsies. Myofibers of controls showed a VPS15 puncta staining that was particularly evident in glycolytic type II fibers (Figure 4,Supplementary Fig. S3). This pattern was completely disrupted in the DD male patients (pts. 1, 2 and 3) but preserved in the less affected DD female pt. 4 (Figure 4). Similarly, the puncta staining became more diffuse in GSDII myofibers, and especially in atrophic and vacuolated fibers. This localization worsened in the second biopsy but it was partially recovered after ERT (Figure 5). Consistently, both DD and GSDII muscles showed an increased and diffuse VPS15 staining in atrophic/vacuolated fibers (Figures 4 and 5). This VPS15 mislocalization is coherent with the hypothesis that VPS15 is not part of a functional PI3K complex and that this abnormal localization may contribute to alter not only autophagosome delivery but also endosome trafficking and lysosome biogenesis/maturation.

To confirm this hypothesis we monitored GAA processing in DD muscles, which present glycogen accumulation similar to GSDII but normal GAA activity.^11^ The maturation steps of GAA start with the synthesis of a 110 kDa inactive precursor in the endoplasmic reticulum (ER) that is cleaved to an intermediate 95 kDa immature form. This 95 kDa GAA is further processed to generate two active proteins of 76 and 70 kDa in the lysosomes. This maturation process happens during the trafficking from ER, to Golgi, multivesicular bodies, late endosomes and lysosomes. Abnormalities in the vesicular trafficking would results in an impairment of GAA maturation with accumulation of intermediate immature forms. We checked the different GAA forms in DD patients as readout of the status of vesicular trafficking. Interestingly, the 110 kDa precursor and the 95 kDa immature form were significantly increased in pts. 1 and 2 (Figure 3a and e) who showed the highest and abnormally localized VPS15 protein (Figure 3a and c and Figure 4). This finding further suggests that vesicular trafficking is altered when VPS15 is accumulated and mislocalized.

### TFEB is differentially activated in DD and GSDII patients

**Figure 6:**
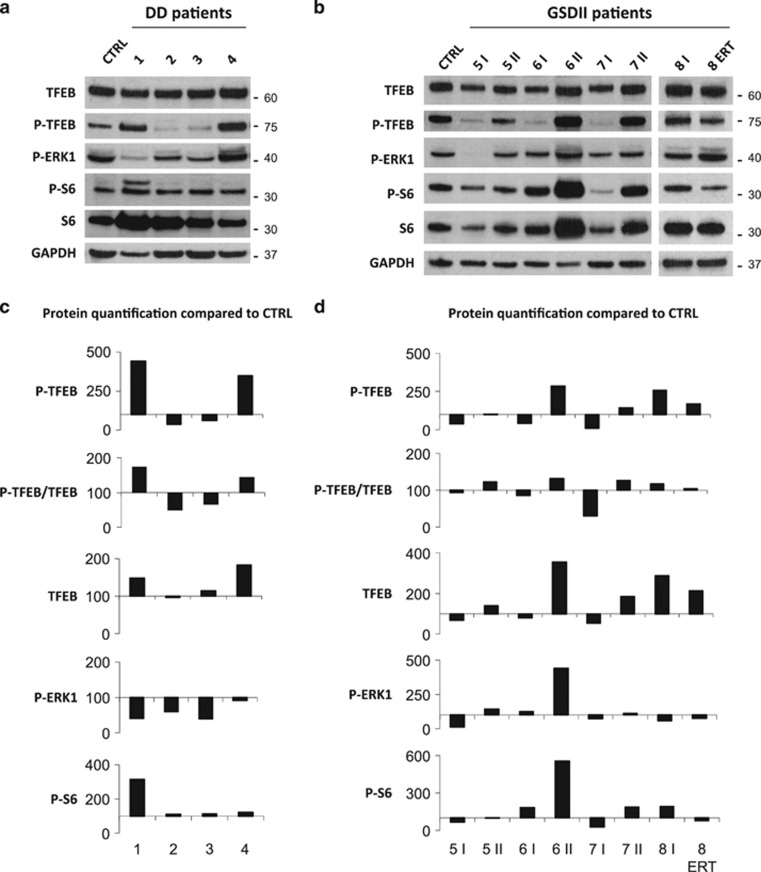
Analysis of TFEB and its regulation in DD and GSDII patients. (**a**) Immunoblot analysis of muscle biopsies for TFEB, P-TFEB, P-ERK1, P-S6, S6 and the loading control GAPDH in DD and in GSDII (**b**) patients. Ctrl, healthy control; ERT, biopsy after ERT; numbers, patient identifiers; I, first biopsy; II, second biopsy. (**c**) Densitometric analysis (percentage compared with control) of proteins normalized to GAPDH for DD and GSDII (**d**) patients

Since GAA maturation is abnormal in DD, we monitored the expression and localization of TFEB, the master regulator of the 'CLEAR' (coordinated lysosomal expression and regulation) gene network.^21^ *In vitro* TFEB overexpression results in upregulation of lysosomal genes, lysosome expansion,^22^ exocytosis and autophagy.^23^ TFEB localizes in the cytoplasm when is phosphorylated by mTORC1 complex and ERK1/MAPK1 kinase, but translocates to the nucleus after dephosphorylation via Calcineurin.^1, 24, 25^ TFEB phosphorylation status by immunoblotting showed some heterogeneity in DD: pts. 2 and 3 showed a dephosphorylated TFEB, while pts. 1 and 4 showed a hyperphosphorylated TFEB when compared with controls (Figure 6a and c).

In GSDII muscles we found that TFEB was more phosphorylated (P-TFEB) and therefore inhibited during the progression of the disease. Indeed, increased P-TFEB levels were found in the second biopsies of GSDII patients with the exception of pt. 8, in which P-TFEB was decreased after ERT treatment (Figure 6b and d). Interestingly, pt. 7 showed the strongest inhibition of TFEB and was the one with the greatest deterioration of muscle force, the largest accumulation of p62-positive inclusions and the most important fiber atrophy.^14^ Indeed the first biopsy of pt. 7, which had an almost normal autophagy,^14^ showed an important TFEB dephosphorylation, while the second biopsy, showing an important autophagy impairment and fiber atrophy,^14^ presented TFEB phosphorylation and inhibition. Conversely, pt. 5, which had the weakest TFEB inhibition, showed a milder disease progression, with slightly increased atrophy and only 2% of p62-positive fibers. These data suggest that disease progression and autophagy impairment are associated with TFEB phosphorylation in GSDII but not in DD.

**Figure 7:**
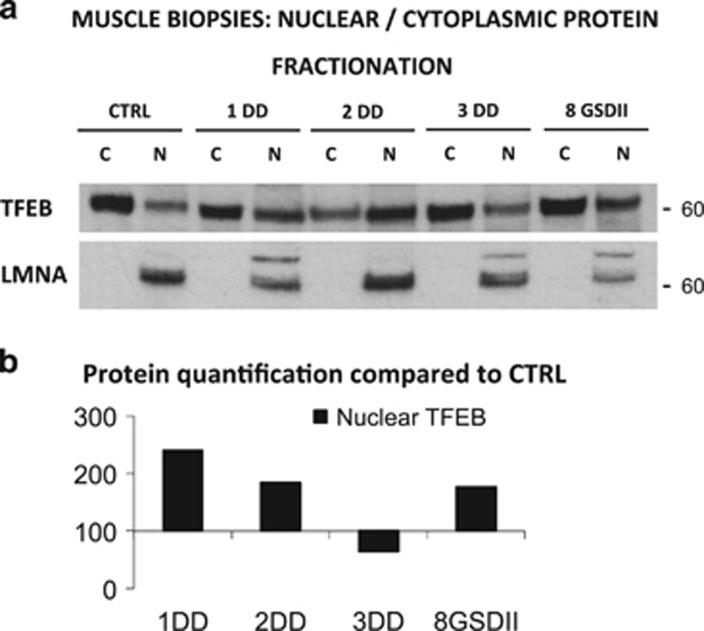
TFEB in nuclear and cytoplasmic protein fractions from muscle biopsies. (**a**) Immunoblot analysis of nuclear and cytoplasmic TFEB. GAPDH was used as loading control for the cytoplasmic protein fraction and LMNA for the nuclear protein lysate. Ctrl, healthy control; numbers, patient identifiers. C, cytoplasmic protein fraction; N, nuclear protein fraction. (**b**) Densitometric analysis (percentage compared with control) of nuclear TFEB normalized to LMNA

As TFEB phosphorylation occurs on the surface of lysosomes and is mTORC1 dependent,^25^ we monitored the level of phosphorylation of RPS6/S6, a downstream target of mTOR. However, the match between TFEB phosphorylation and mTORC1 activity was absent in DD (Figure 6a and b), while it was conserved in GSDII patients (Figure 6c and d). As TFEB is also controlled by ERK1/MAPK1 kinase, we monitored the activation of this pathway. Interestingly, ERK1 phosphorylation was reduced in DD, while it was increased in the second biopsies of GSDII. Therefore, the changes of P-TFEB result from the combination of mTOR and ERK1 activities. To determine whether TFEB phosphorylation status reflects nuclear relocalization we purified the nuclear and cytosolic fraction of muscle biopsies, but we could get enough material only from DD pts. 1, 2 and 3 and GSDII pt. 8 before the ERT treatment. An enrichment of nuclear TFEB was detected in the DD pts 1 and 2, and in the GSDII pt. 8 when compared with controls (Figure 7).

**Figure 8:**
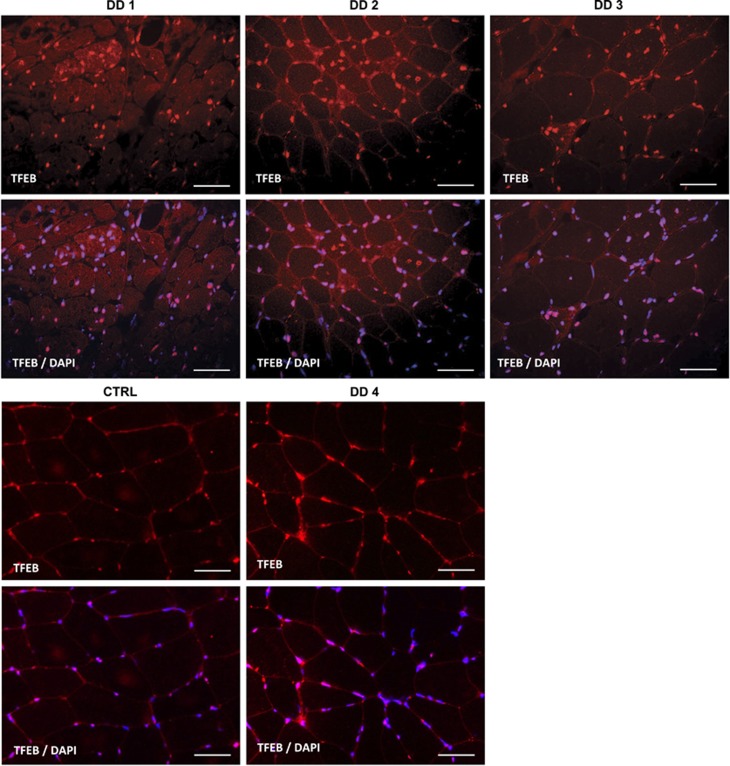
TFEB localization in DD. Immunofluorescence for TFEB (red) and DAPI (blue) show increased activation (nuclear translocation) of TFEB in male patients (pts. 1–3) compared with female (pt. 4) and healthy control (Ctrl). Bar=40 *μ*m

**Figure 9:**
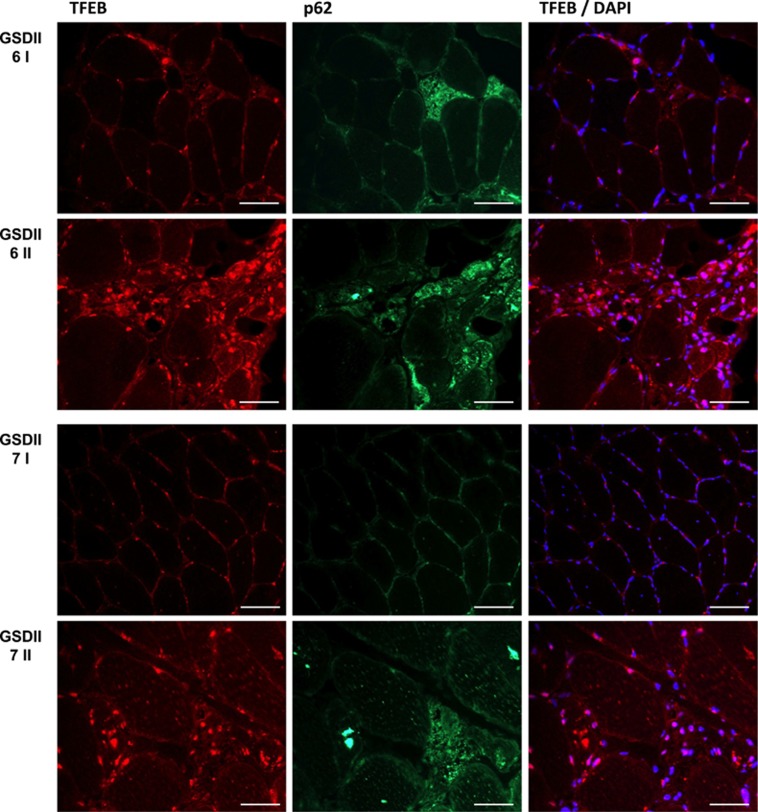
TFEB localization in two untreated GSDII patients after 6 years of disease progression. The upper panel shows the first (I) and the second (II) biopsy of patient 6, the lower panel shows the first (I) and the second (II) biopsy of patient 7. Immunofluorescence for TFEB shows increased activation in atrophic/autophagy-incompetent fibers, which correlates with disease severity. Bar=40 *μ*m

As disease severity and autophagy impairment may impact on TFEB nucleus/cytosol shuttling, we checked whether TFEB localization was affected by the autophagosomes buildup. We found that TFEB was mostly localized in nuclei in DD male patients (Figure 8) but not in GSDII, who were more heterogeneous (Figure 9): TFEB was translocated into the nuclei of fibers that were atrophic and p62-positive (Figure 9), but these fibers expressed also higher levels of cytoplasmic TFEB.

By staining serial muscle sections of DD patients for VPS15, CAV3, TFEB, p62, DAPI and neonatal myosin heavy chain (MYH8) we showed that atrophic/vacuolated fibers also displayed upregulation and mislocalization of VPS15, together with increased nuclear TFEB localization and accumulation of p62-positive inclusions (Supplementary Figures S4–S6). Lack of MYH8 expression showed that these fibers are atrophic and not regenerating.

**Figure 10:**
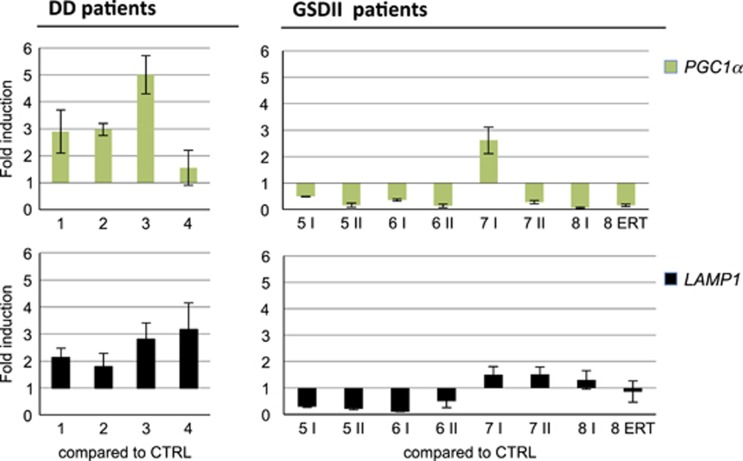
qPCR analysis of *PGC1α* and *LAMP1* in DD and GSDII muscle biopsies. Ctrl, healthy control; numbers, patient identifiers; I, first biopsy; II, second biopsy; ERT, biopsy after ERT

To confirm the different regulation of *TFEB* we monitored the expression of two downstream targets, *PPARGC1A/PGC1α* and *LAMP1*. In agreement with the prevalent nuclear localization in DD patients, we found that TFEB targets were mostly induced in DD and suppressed in GSDII patients when compared with controls (Figure 10).

## Discussion

AVMs are a group of muscle disorders characterized by massive autophagic buildup, but whether this buildup is an autophagy enhancement or impairment remains unclear. In GSDII autophagy impairment has been demonstrated to contribute to muscle pathology both in a mice model (GAA-ko)^13^ and in patients.^14^ In this study we assessed whether autophagy impairment is a common pathogenetic mechanisms between two AVMs: DD and GSDII. We showed that, similar to GSDII, DD muscles display an autophagy impairment that correlates with disease severity. Notably, the accumulation of autophagosomes affects also cellular trafficking and signaling. Indeed, autophagy is a fundamental homeostatic pathway that feed lysosomes but also other lysosome-merging routes, such as endocytic, biosynthetic-secretory and retrieval pathways. We analyzed also some key factors involved in cellular trafficking and autophagy regulation. All patients showed an increased amount of the VPS15 protein, which correlated with disease severity. However, this increase resulted in an abnormal localization of VPS15 protein, which might alter the normal PI3P production by the PI3K complex. This hypothesis is sustained by the phenotype of muscle-specific VPS15 knockout mice that reproduces many features of DD muscles.^19^ Furthermore, the finding of an impaired GAA processing in DD patients strongly supports the concept of an altered endosome trafficking and lysosome maturation that results in autophagosome buildup.

Similarly to GSDII, we showed that the impairment of autophagy in DD contributes to muscle loss. However, looking at the expression of the critical atrophy-related genes we found a significant induction of *ATROGIN1* and *BNIP3* but not of *MURF1*: *MURF1* was induced in DD pt. 2 but not in the severely affected DD pt. 1. This might be explained by the fact that *MURF1* induction precedes muscle atrophy and returns to baseline in atrophic muscles. Therefore, *MURF1* expression is unchanged in pt. 1 because his muscles are already severely atrophic.

The analysis of TFEB highlighted some differences between DD and GSDII patients. DD patients showed TFEB nuclear translocation and upregulation of *LAMP1* and *PGC1α* genes, two well-known TFEB downstream targets. Conversely, GDSII patients showed a correlation between disease progression and TFEB inhibition. This finding is supported also by the fact that mTORC1 and ERK1 were induced in the second GSDII biopsies and that TFEB target genes were generally suppressed. However, looking at TFEB localization, we found a nuclear staining in p62-positive fibers. This nuclear relocalization of TFEB in autophagy impaired fibers might reflect the last chance to re-activate lysosomal-dependent degradation via a transcriptional program. In GSDII, the discrepancy between localization and phosphorylation might result from the contribution of unaffected fibers. Alternatively, besides mTORC1 complex and ERK1, other factors might regulate TFEB localization or activity in GSDII. The fact that pt. 8 showed an enrichment of nuclear TFEB but only minor effects on gene expression suggests that other nuclear factors regulate TFEB recruitment and action on target promoters.

Our results show the limitation of our understanding about the pathogenesis of muscle damage in AVMs. For years, the muscle loss in GSDII has been simply attributed to enlargement of glycogen-filled lysosomes followed by lysosomal rupture. This scenario is an over simplification that takes into account the morphological features of GSDII muscles. However, the recent data showing that lysosomes are a signaling hub^25^ completely change this picture. Indeed, we showed that lysosomal dysfunction has a great impact not only in cellular trafficking such as autophagosome/endosome maturation and delivery, but also in regulation of important signaling pathways such as mTOR, ERK1 and TFEB. We have previously demonstrated that autophagy reactivation is beneficial in GSDII because it reactivates the endosome trafficking and, consequently, the maturation of GAA, the uptake of rGAA and the delivery to lysosomes. We showed that VPS15, VPS34 and BECN1 proteins are altered in DD and GSDII. These proteins belong to the PI3K complex that is crucial for autophagosome formation and endosome trafficking. Therefore, the VPS15/VPS34/BECN1 changes in DD and GSDII fits with the hypothesis of an impaired endosome and lysosomal maturation resulting in autophagosome buildup. Interestingly, studies in yeast have suggested the requirement of Vps34 in the retrograde trafficking from endosomes to Golgi,^26^ which includes the recycling of sorting receptors, such as the cation independent mannose-6 phosphate receptors involved in GAA trafficking. Importantly, there are drugs, such as Tat-Beclin1, that targets BECN1 complex, and that might be used in these patients to restore autophagy, ameliorate muscle atrophy and improve the delivery to lysosomes of rGAA. Finally, we showed that lysosomal dysfunction affected the localization and activity of TFEB. As we have recently found that TFEB controls glucose homeostasis and mitochondrial biogenesis,^27^ it is possible that TFEB-mediated metabolic changes contribute to disease progression in AVMs. Therefore, modulation of mTOR-TFEB axis might also be considered as potential therapeutic approach. In conclusion, autophagy impairment is a common pathogenetic mechanism in AVMs, which affects also cellular signaling. A residual lysosomal activity seems to be crucial for maintenance of autophagy and autophagy-regulating factors, which have a role in disease outcome. Therapeutic approaches, which may also include exercise and nutritional intervention, targeted to normalize these factors and restore the autophagic flux in these patients, should be considered.

## Materials and methods

### Patients and muscle biopsies

We selected four DD patients (three males, one female) and four GSDII adult patients (one male, three females; Supplementary Table S1) and diagnosed following clinical examination, muscle biopsy histopathology and identification of mutations in the *LAMP2* and *GAA* gene, respectively. The four GSDII patients are the same involved in a previous study.^14^ For each GSDII patient we analyzed two muscle biopsies, which have been obtained in one case (pt. 8) before and after ERT and in the other cases after 6 (pts 6, 7) and 9 (pt.5) years of disease progression. Muscle biopsies of patients and healthy controls were obtained from the Telethon Biobank network. Experiments were approved by the local ethics committee at the University of Padova. We selected age- and sex-matched controls (six females, aged 32–54 years; three males, aged 18–28 years) for the morphometric analysis and adult controls for the immunoblotting and gene expression analyses.

### Nuclear/cytoplasmic protein fractionation

A variable number (from 50 to 100) of fresh-frozen sections (10 *μ*m thick) of muscle biopsies were lysed in the NE-PER Nuclear and Cytoplasmic Extraction Kit buffers following manufacturer's instructions (Life Technologies, Milan, Italy). Proteins were then solubilized in lysis buffer for western blotting, as described below.

### Immunoblotting

Thirty 10 *μ*m-thick fresh-frozen sections of muscle biopsies were lysed in a buffer containing 50 mM Tris, pH 7.5, 150 mM NaCl, 10 mM MgCl_2_, 0.5 mM DTT, 1 mM EDTA, 10% glycerol, 2% SDS, 1% Triton X-100 and protease inhibitors (Complete Protease Inhibitor Cocktail, Roche, Basilea, Switzerland). The samples were immunoblotted as previously described^28^ and visualized with the chemiluminescent substrate ECL (GE Healthcare, Milan, Italy). When necessary, we stripped and reprobed the membranes. The stripping buffer consisted of 25 mM glycine-HCl, pH 2, and 1% SDS. GAPDH was used as loading control. We carried out densitometric quantification from multiple gels for each experiment using the ImageJ software (US National Institutes of Health). Values were normalized to the loading control (GAPDH for total and cytoplasm-fraction lysates and LMNA for nuclear fractions).

### Antibodies

Primary antibody to LC3 (L7543) was from Sigma-Aldrich (St. Louis, MO, USA). Antibody to p62 (Gp62-C) was from Progen Biotechnik GmbH (Heidelberg, Germany). Antibody to LAMP2 (H4B4) was from Developmental Studies Hybridoma bank (Iowa City, IA, USA)). Antibody to CAV3 (610421) was from Beckton Dickinson Transduction Lab. (Milan, Italy). Antibodies to S6 (2217), P-S6 (Ser240/244) (2215), P-ERK1 (9106), VPS34 (4263) and for western blot analysis of TFEB (4240) were obtained from Cell Signalling (Milan, Italy). Antibody to TFEB (MBS120432) for immunofluorescence was from MyBiosource (San Diego, CA, USA). Antibody to P-TFEB (specific to the Ser142 phosphorylation site) was obtained from Dr. C. Settembre (TIGEM, Naples, Italy). Antibody to VPS15 (H00030849-M02) was from Abnova (Taipei, Taiwan). Antibody to BECN1 (612113) was from BD Transduction Laboratories (Milan, Italy). Antibody to GAA was provided by Sanofi-Genzyme Corporation (Modena, Italy). Antibody to GAPDH (8245) was from Abcam (Cambridge, UK), antibody to LMNA (NCL-LAM-A/C) was from Leica Biosystems (Wetzlar, Germany), antibody to neonatal myosin heavy chain or MYH8 (MHC-n) was from Novocastra (Newcastle upon Tyne, UK), antibody to myosin binding protein C, slow type (MYBPC1) was from Abcam. HRP-labeled secondary anti-mouse (NA931V) and anti-rabbit (NA934V) antibodies were from GE Healthcare; anti-guinea pig (A5545) was from Sigma-Aldrich (St. Louis, MO, USA).

### Immunofluorescence and morphometry

For immunofluorescence (IF), muscle cryosections were collected on Superfrost slides, fixed with 4% paraformaldehyde (PFA), treated with 0.1% Triton, incubated in blocking solution (0.5% BSA, 10% horse serum in PBS) for 20 min, and then incubated overnight at 4 °C with antibodies. Appropriate secondary fluorescent antibodies (Alexa-Fluor, Invitrogen, Paisley, UK) were used. Slides were mounted using Vectashield medium with DAPI stain (Vector, Burlingame, CA, USA) and examined on fluorescence microscope (Leica DM5000B, Wetzlar, Germany).

We calculated the percentage of vacuolated fibers (defined as those presenting either diffuse or scattered intracytoplasmic vacuoles), and of p62-positive fibers (defined as those presenting either diffuse or scattered intracytoplasmic staining). Fiber cross-sectional area (CSA) was measured using ImageJ software. For the patients and controls, we calculated the total mean CSA by measuring all muscle fibers in the examined sections (at least 200 fibers), the mean CSA of fibers with and without p62-positive protein aggregates and the mean CSA of vacuolated and non-vacuolated fibers. Mean reference fiber CSA in healthy controls was 2419±786 *μ*m^2^ for women (*n*=6, 32–54 years) and 3446±950 *μ*m^2^ for men (*n*=3; 18–28 years).

### Gene expression analyses

Total RNA was isolated from muscle biopsies with the Trizol reagent and treated with DNase I (Invitrogen, Paisley, UK) according to the manufacturer's protocol. The yield and purity of the extracted total RNA were determined using a Nanodrop spectrophotometer (Thermo Fisher Scientific, Waltham, MA, USA). cDNA was generated with the iScript cDNA Synthesis Kit (Bio-Rad, Hercules, CA, USA) and analyzed by real-time PCR on an MJ Mini Opticon Thermal Cycler (Bio-Rad) using the IQ SYBR green super mix. Primers specific for *BNIP3* (RefSeq NM_ 004052), *BECN1* (RefSeq NM_ 003766), *p62* (RefSeq NM_003900), *VPS15* (NM_003793), *PGC1α* (NM_013261), *LAMP1* (NM_005561) were designed with the Primer3 software (http://frodo.wi.mit.edu/primer3/). Previously published primer sequences were used for *ATROGIN1*, *MURF1/TRIM63* and *GAPDH*.^29, 30^ Reactions were run in triplicate. Results were normalized to *GAPDH* using the 2^−ΔΔCT^ calculation method. They are expressed as a percentage of the healthy control and are plotted as mean±S.D.

### Statistical analysis

Data were expressed as means±standard deviation. Differences between groups were assessed using analysis of variance (Student's unpaired *t* test) and linear regression analysis. We considered a *P*-value <0.05 to be significant.
