# Supplementary material for: Autophagy dysregulation in Danon disease
Source: Cell Death Dis. 2017 Jan 19;8(1):e2565–. doi: 10.1038/cddis.2016.475 (PMC5386379; doi:10.1038/cddis.2016.475)
Supplement: Supplementary Table S1 [file cddis2016475x1.pdf]

Supplementary Material for “Autophagy dysregulation in Danon Disease”

Anna Chiara Nascimbeni[1,2], Marina Fanin[1], Corrado Angelini[3], Marco Sandri[4,5]

[1]Department of Neurosciences, University of Padova, Biomedical Campus Pietro d’Abano, Padova, Italy; [2]Institut Necker Enfant-Malades (INEM), INSERM U1151-CNRS UMR 8253, [2]Université Paris Descartes-Sorbonne Paris Cité, Paris, France; [3]Fondazione IRCCS San Camillo Hospital, Venice, Italy; [4]Dulbecco Telethon Institute at Venetian Institute of Molecular Medicine, Padova, Italy, [5]Department of Biomedical Science, University of Padova, Italy

*Corresponding author: e-mail: marco.sandri@unipd.it, tel: +39 049 7923258, fax: +39 049 7923250

Supplementary Figures S1-S6

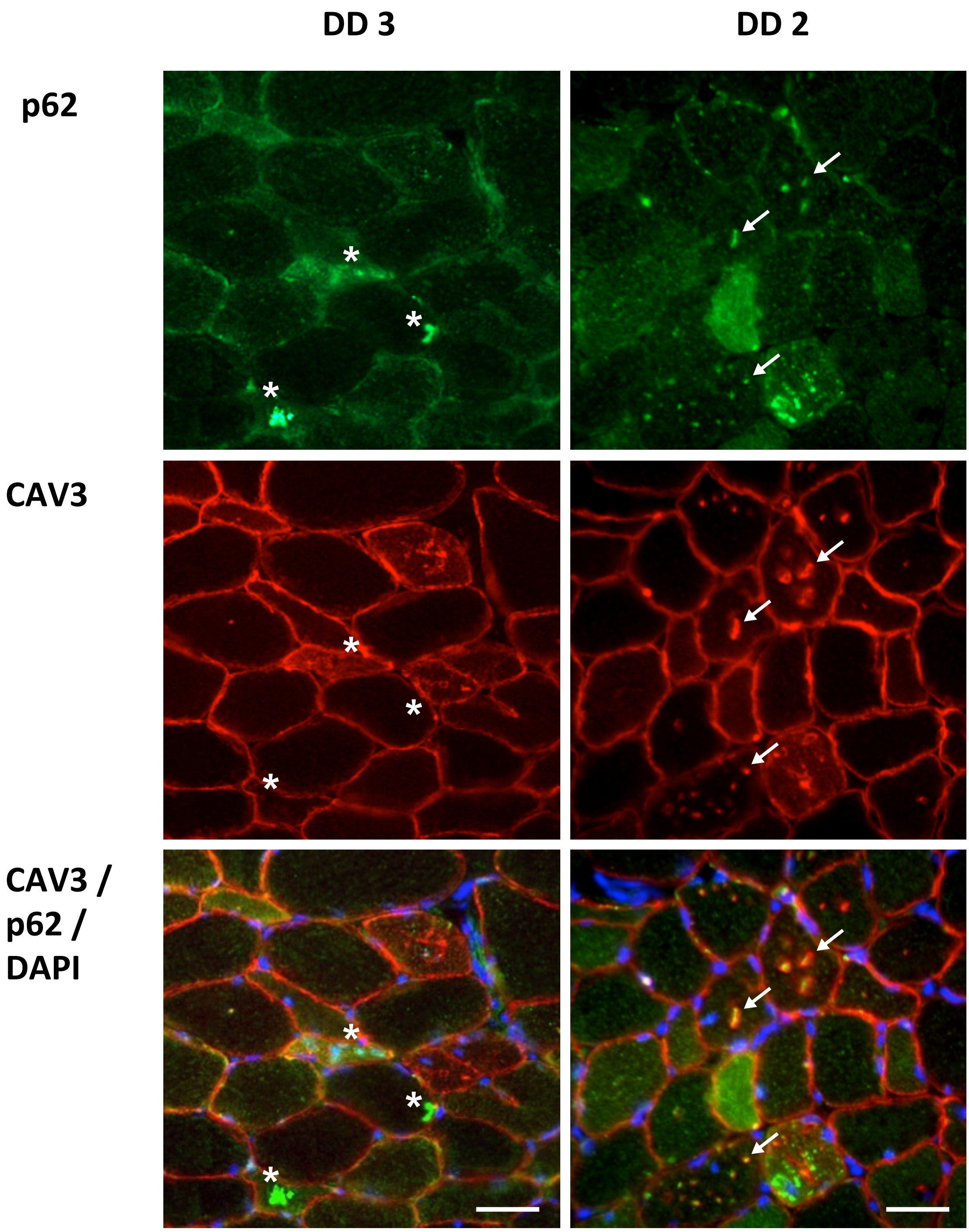

Figure S1. Example of different p62-positive protein aggregates in muscle biopsies of DD patients. p62-positive protein aggregates (green) are free in the cytoplasm (asterisks) or entrapped in vacuoles with sarcolemmal features (AVSF) (arrows). Membranes are labelled with CAV3 (red) and nuclei are labelled with DAPI (blu). Bar = 40 µm.

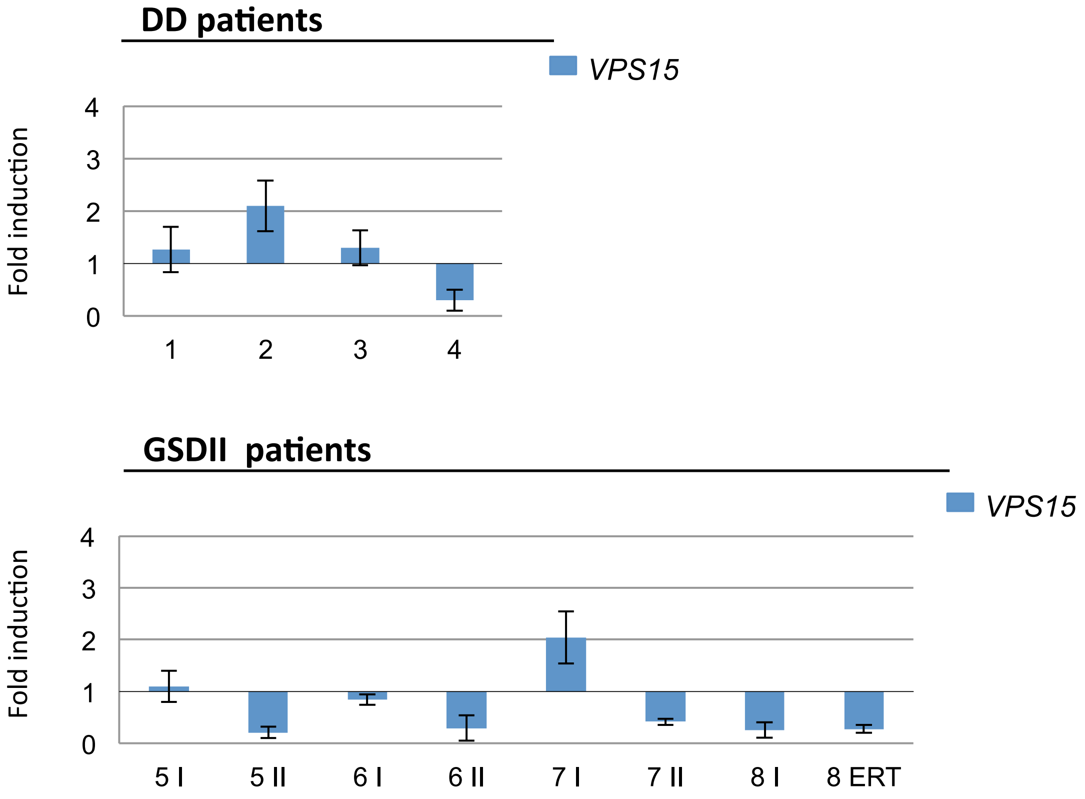

Figure S2. qPCR analysis of *VPS15* in muscle biopsies from DD and GSDII patients. DD patients (1-4), GSDII patients (5-8, I: first biopsy, II: second biopsy, ERT: enzyme replacement therapy).

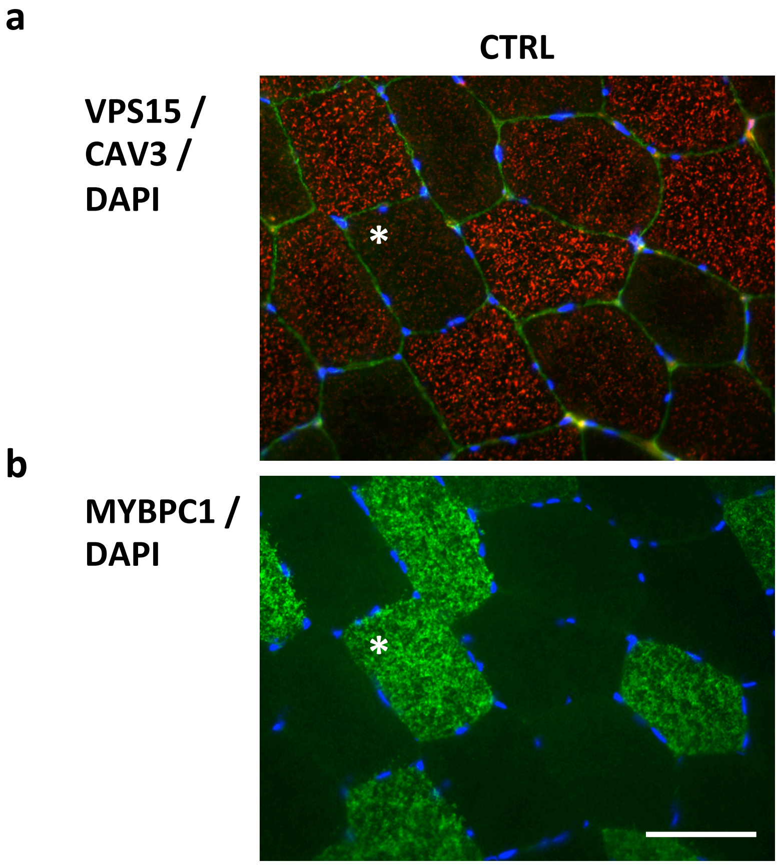

Figure S3. VPS15 distribution in skeletal muscle. Serial sections of muscle fibers from a healthy control (CTRL) are immunostained for (**A**) VPS15 (red), CAV3 (green) and DAPI (blu), and (**B**) for myosin binding protein C, slow type (MYBPC1) (green) and DAPI (blu). VPS15 is expressed mainly by type II fast twitch fibers. Asterisks label the same fiber in different sections. Bar = 40 µm.

DD 1

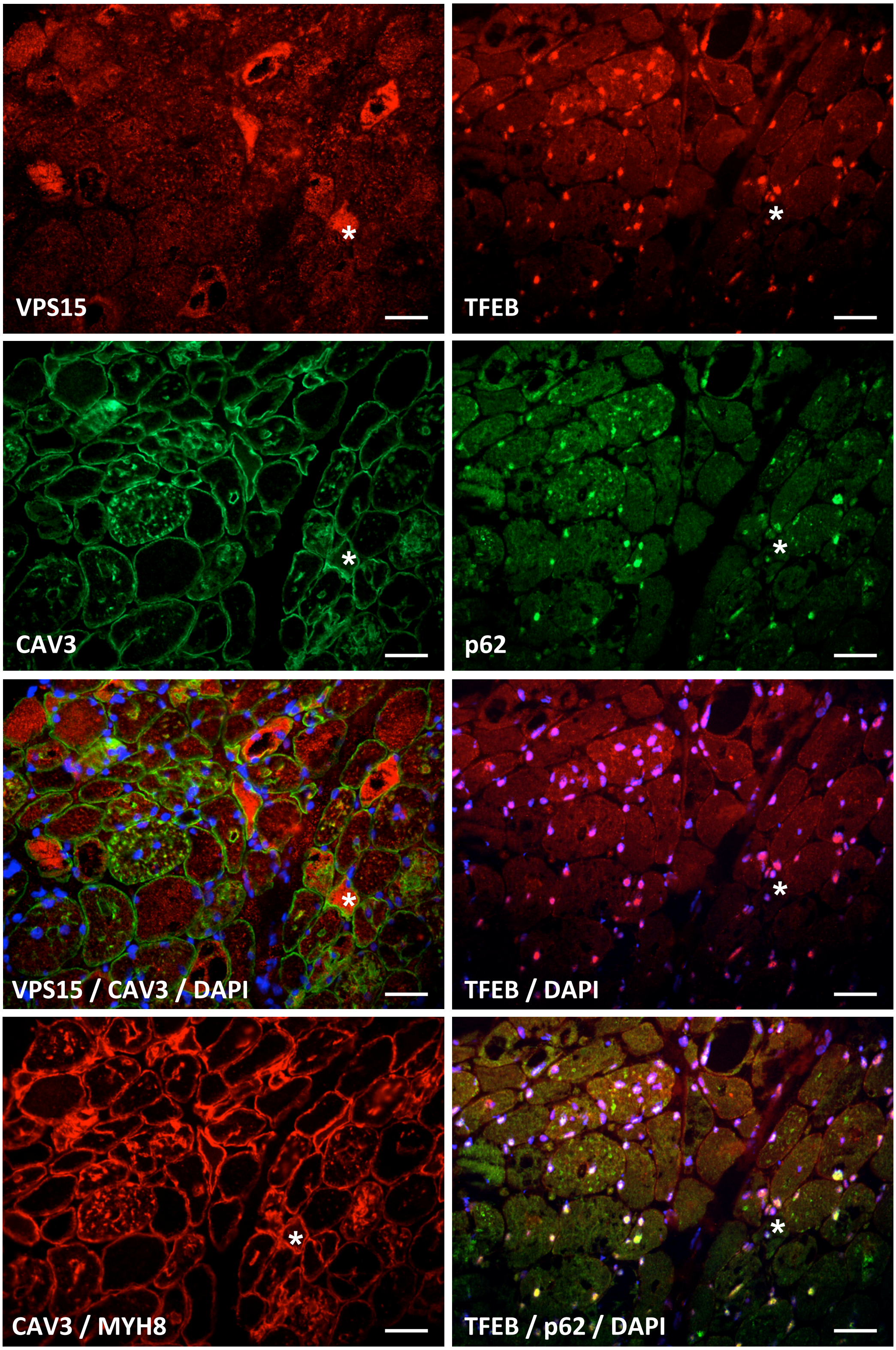

Figure S4. Atrophic fibers show VPS15 accumulation/mislocalization, TFEB activation and p62-positive protein aggregates. Serial sections of muscle biopsy from DD patient 1, immunostained for VPS15 (red), CAV3 (green) and DAPI (blu), TFEB (red), p62 (green), MYH8 (red). Asterisks show the same atrophic fiber in the different serial sections. Bar = 40 μm.

DD 2

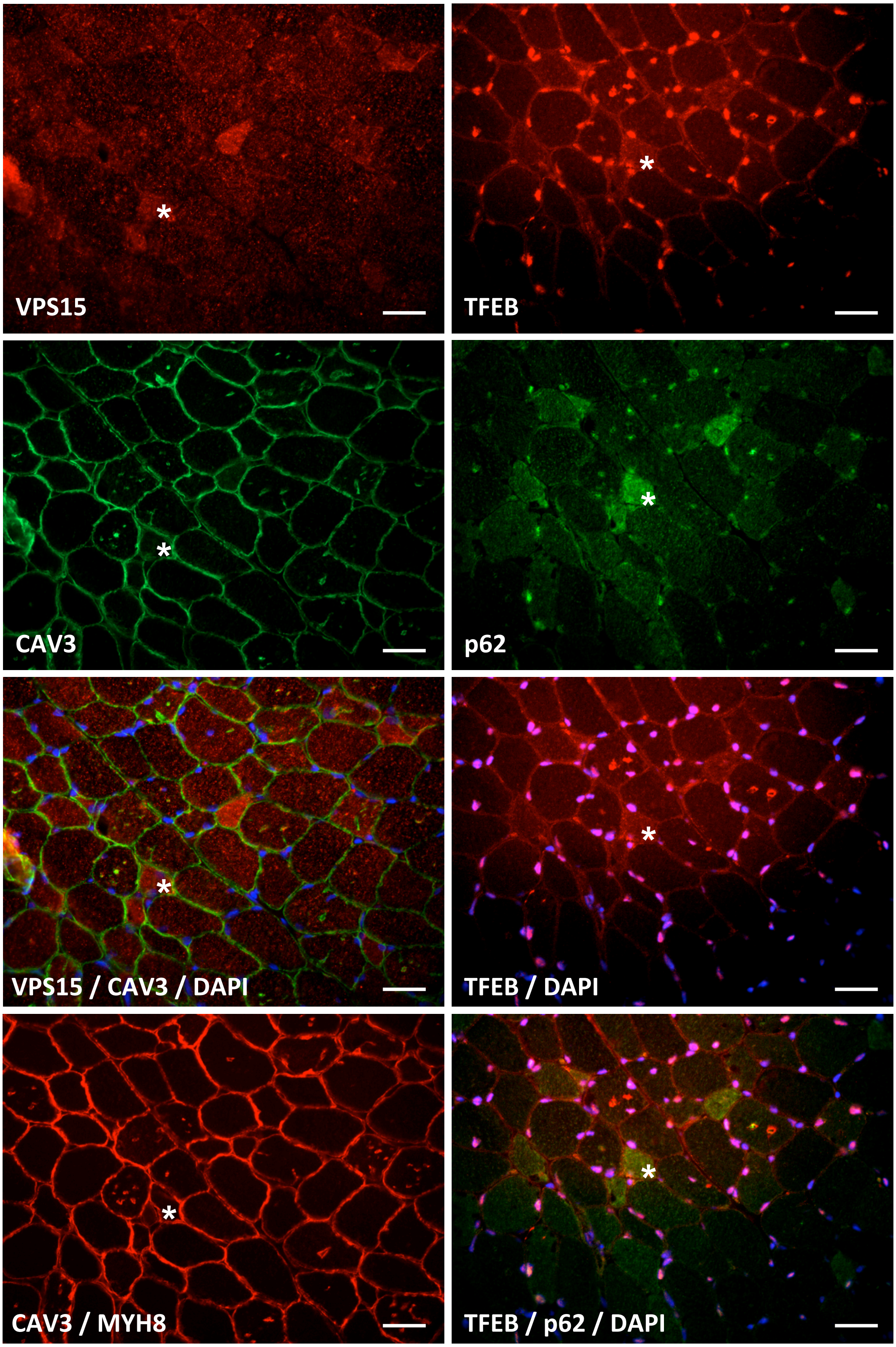

Figure S5. Atrophic fibers show VPS15 accumulation/mislocalization, TFEB activation and p62-positive protein aggregates. Serial sections of muscle biopsy from DD patient 2, immunostained for VPS15 (red), CAV3 (green) and DAPI (blu), TFEB (red), p62 (green), MYH8 (red). Asterisks show the same atrophic fiber in the different serial sections. Bar = 40 µm.

DD 3

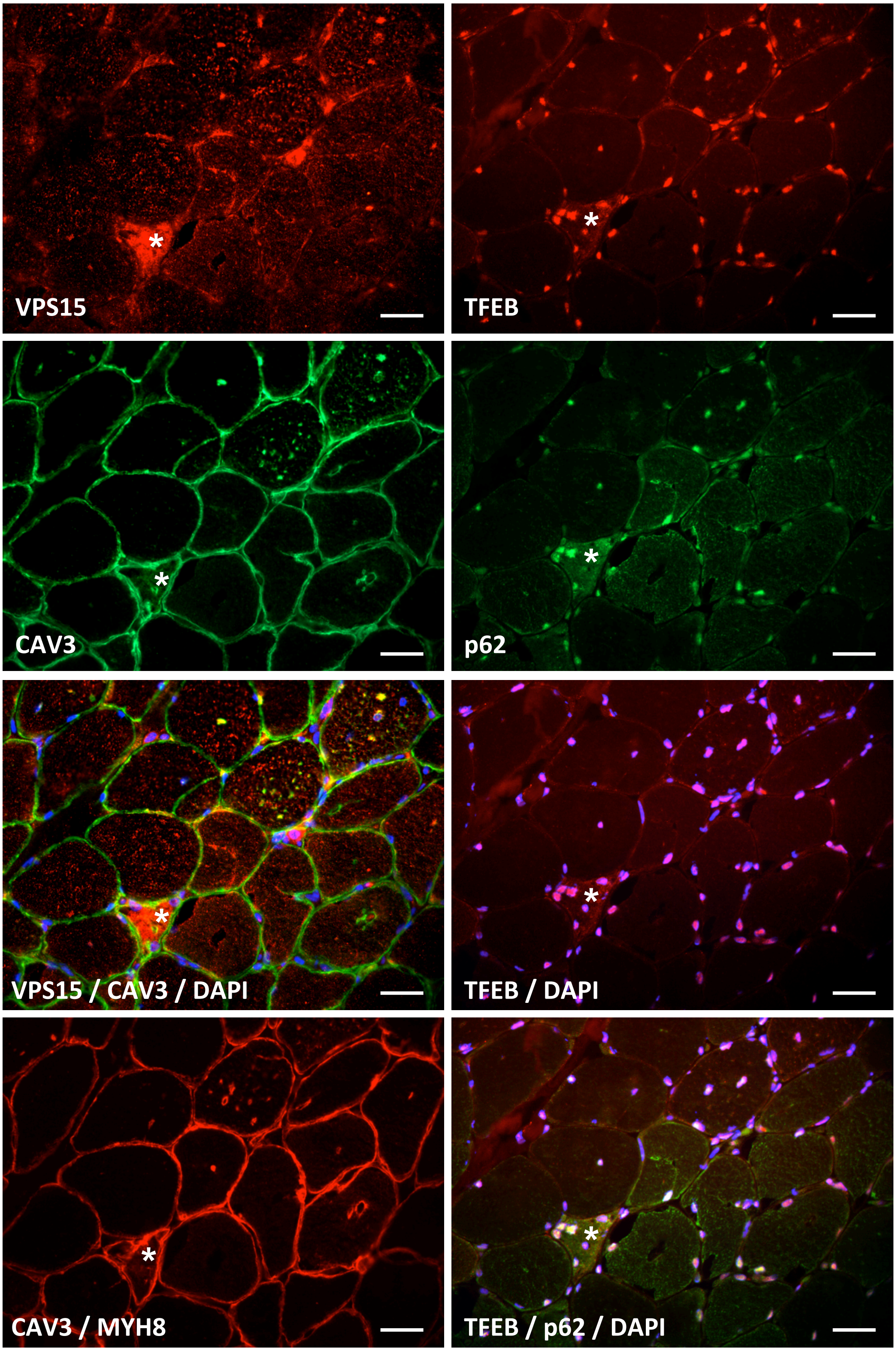

Figure S6. **Atrophic fibers show VPS15 accumulation/mislocalization, TFEB activation and p62-positive protein aggregates.** Serial sections of muscle biopsy from DD patient 3, immunostained for VPS15 (red), CAV3 (green) and DAPI (blu), TFEB (red), p62 (green), MYH8 (red). Asterisks show the same atrophic fiber in the different serial sections. Bar = 40 µm.
