# Supplementary material for: Autophagy dysregulation in Danon disease
Source: Cell Death Dis. 2017 Jan 19;8(1):e2565–. doi: 10.1038/cddis.2016.475 (PMC5386379; doi:10.1038/cddis.2016.475)
Supplement: Supplementary Information [file cddis2016475x2.pdf]

Table S1. Patients' clinical and molecular data

Patient		Gender	Age at onset	Age at biopsy	Disease duration	Main clinical features at biopsy	Degree of clinical severity	Genotype
1 DD		M	20	21	1	Severe hypertrophic cardiomyopathy, severe muscle impairment and atrophy, cardiogenic ascites, cachexia, deceased at age 21	++++	c.796–797insC, p.fsX7
2 DD		M	22	25	3	Severe hypertrophic cardiomyopathy, moderate muscle impairment, deceased at age 25	+++	c.680–701del22, p.fsX8
3* DD		M	18	28	10	Severe hypertrophic cardiomyopathy, moderate muscle impairment, heart transplant at age 34, alive at age 40	++	c.294G>A, p.W98X
4* DD		F	26	54	28	Severe hypertrophic cardiomyopathy, mild muscle impairment, heart transplant at age 52, alive at age 61	+	c.294G>A, p.W98X
5 GSDII	I	F	40	56	16	Moderate muscle weakness, waddling gait	++	c.-32-13T>G; c.307T>G, p.C103G
	II			65	25	Severe muscle weakness, walks with a cane, dyspnea	+++	
6 GSDII	I	F	34	54	20	Moderate muscle weakness, Gowers sign	++	c.-32-13T>G; c.2219delTG, p.V740fsX55
	II			60	26	Severe muscle weakness, respiratory insufficiency	+++	
7 GSDII	I	F	41	42	1	Mild muscle weakness, Gowers sign	+	c.-32-13T>G; c.546+1G>T
	II			48	7	Moderate muscle weakness, loss of Gowers	++	
8 GSDII	I	M	18	18	0	Asymptomatic hyperCKemia (>1000 U/L)	+/-	c.546+1G>T, p. L552P; n.d.
	ERT			19	1	Asymptomatic hyperCKemia (>700 U/L)	+/-	

I: first biopsy; II: second biopsy; *: relatives, son and mother; CK: creatine kinase (normal values = 0-200 U/L); n.d.: not determined.
